# Blackcurrant (Fruits, Pomace, and Leaves) Phenolic Characterization before and after In Vitro Digestion, Free Radical Scavenger Capacity, and Antioxidant Effects on Iron-Mediated Lipid Peroxidation

**DOI:** 10.3390/foods13101514

**Published:** 2024-05-13

**Authors:** Arabela Elena Untea, Alexandra-Gabriela Oancea, Petru Alexandru Vlaicu, Iulia Varzaru, Mihaela Saracila

**Affiliations:** Feed and Food Quality Department, National Research and Development Institute for Biology and Animal Nutrition, Calea Bucuresti, No. 1, 077015 Balotesti, Romania; alexandra.oancea@ibna.ro (A.-G.O.); alexandru.vlaicu@ibna.ro (P.A.V.); iulia.maros@ibna.ro (I.V.); mihaela.saracila@ibna.ro (M.S.)

**Keywords:** blackcurrant, byproducts, digestibility, polyphenols, antioxidant activity

## Abstract

Blackcurrant (*Ribes nigrum* L.) is a berry bush widely cultivated in Europe for producing juices, jams, jellies, and syrups. In addition to berries, blackcurrant leaves and pomace, as byproducts, have also been shown to have health-promoting effects. Static digestion, simulating oral, gastric, and small intestinal digestion, was applied, and blackcurrant leaves, fruits, and pomace and the polyphenol bioaccessibility were evaluated in terms of recovery index. The results were related to sample type, and the recovery index presented higher values in the case of fruits, indicating this morphological part of blackcurrant as the most bioaccessible. The antioxidant potential of blackcurrant was evaluated using four different methods, with the leaves proving to be a significant and powerful antioxidant compared to fruits and pomace. The counteracting potential of inhibiting the oxidation process was evaluated using in vitro-induced lipid peroxidation and the inhibition potential of superoxide and hydroxyl anions. The antioxidant evaluation and the inhibition of biological and non-biological radicals indicate the leaf extract is the most powerful antioxidant studied. Also, the results proved that not only fruits but also the blackcurrant byproducts (pomace and leaves) are promising sources of bioaccessible antioxidants with potential benefits in animal nutrition.

## 1. Introduction

Berries are colored fruits belonging to the genera Rubus (blackberries, raspberries), Vaccinium (blueberries, cranberries, lingonberries), Fragaria (strawberries), Ribes (blackcurrant), or Lycium (goji berries) [1]. They are known for their health-promoting properties, like chemopreventive and chemotherapeutic effects [2]. Berries are known to be a rich source of polyphenols, particularly anthocyanins, a class of flavonoids responsible for the color of fruits [3]. Among these genera of berries, blackcurrant (*Ribes nigrum*) stands out as an underutilized food resource, renowned for its abundance of antioxidants. In Romania, it is primarily harvested by local inhabitants, yet its economic significance remains modest. The fruits are consumed in fresh form or as juices, jams, jellies, and syrups. The leaves and pomace are valuable raw materials with phenolic concentrations and profiles different from the fruit’s [4]. Some studies found no correlation between polyphenol profiles in different morphological plant parts. In many cases, berry fruits are poor sources of antioxidants compared with leaves [5].

Polyphenols are a class of compounds that present multiple aromatic rings and hydroxyl groups, split into several branches like phenolic acids, flavonoids, lignans, tannins, or stilbenes [6]. Flavonoids are the most abundant polyphenol group in fruits and vegetables and are C6–C3–C6 phenylpropanoids consisting of two phenyl rings (rings A and B) and one heterocyclic ring (ring C). According to the unsaturation degree and the carbon position on ring C, the flavonoids can be subdivided into several groups, like flavones, flavonols, flavanones, anthocyanins, and chalcones [7].

Bioefficiency (implying terms like bioavailability, bioaccessibility, and bioactivity) is a concept found in the scientific literature describing the relationship between foods and their health benefits.

According to [8], bioactivity is a nutrient’s ability to produce a biological effect, bioavailability is described as the quantity of ingested food that reaches the organs and tissues, and bioaccessibility is the fraction of the targeted nutrient that becomes accessible for absorption in the gastrointestinal tract. In other words, bioaccessibility quantifies the nutrient release ratios from the food matrix and their solubilization in oral, gastric, or intestinal fluids. Due to ethical concerns, human studies are avoided, and bioaccessibility can be successfully determined using in vitro digestion models with standardized relevant conditions, as proposed by [9]. The proposed system is currently used to study the bioaccessibility of different classes of polyphenols [10]. The bioavailability of polyphenols depends on their molecular weight. Isoflavones and some phenolic acids, like caffeic acid and gallic acid, present higher absorption rates, followed by catechins, flavanones, and quercetin glucosides. High-molecular-weight polyphenols like anthocyanins and proanthocyanidins are considered molecules with low absorption rates [11]. Studies on phenolic compound reactions during gastrointestinal phases provided evidence of several factors’ effects, like pH, enzymes, peristaltic movements, and food matrix [12].

Reactive oxygen species (ROS), including oxygen free radicals, are byproducts of normal metabolic processes or appear from exogenous sources, but their presence is inhibited by an endogenous antioxidant defense system. When the rate of the antioxidative and prooxidative substances is unbalanced, oxidative stress directly impacts lipids and protein structures [13]. Plant antioxidants, particularly polyphenols, are known to be powerful antioxidants and free radical scavengers.

Animal nutrition is considered to be an important field necessary to obtain food of animal origin, but at the same time, it cannot compete with human nutrition in terms of resources. In fact, the search for new vegetable sources of bioactive compounds with low economic value became a permanent concern for specialists in animal nutrition. The byproducts resulting from the industrial use of berries (pomace or meals), as well as their leaves, represent potential sources of antioxidants for the nutrition of monogastrics, whose composition and bioavailability are rarely reported in the scientific literature. The study of these byproducts in relation to the main part exploited in the food industry (berries) provides scientific data necessary for their use in animal nutrition.

In this context, the study aimed to evaluate the difference between the bioaccessibility and digestive stability of phenolic compounds from different matrices belonging to the same vegetal material (blackcurrant fruits, pomace, and leaves) and to provide comparative data regarding the antioxidant and free scavenging potential of the considered samples.

## 2. Materials and Methods

### 2.1. Chemicals

1,1-Diphenyl-2-picrylhydrazyl (DPPH), butylated hydroxytoluene (BHT), nitro blue tetrazolium dihydrochloride (NBT), phenazine methosulphate (PMS), nicotinamide adenine dinucleotide (NADH), 2-deoxy-D-ribose, hydrogen peroxide, disodium ethylenediaminetetracetate (EDTA), trichloroacetic acid (TCA), ferric chloride, thiobarbituric acid (TBA), and ascorbic acid (AA) were purchased from Sigma (Sigma-Aldrich GmbH, Steinheim is the correct city nameSteinheim, Germany). All other chemicals were of analytical grade.

Phenolic standards: ellagic acid (95%), syringic acid (98%), epicatechin (96%), 4-hydroxy-3-methoxy-cinnamic acid (95%), rutin (95%), vanillic acid (95%), 3-hydroxybenzoic acid (95%), protocatechuic acid (96%), caffeic acid (95%), coumaric acid (98%), epigallocatechin (97%), catechin (95%), quercetin (95%), and resveratrol (99%) were purchased from Sigma (Sigma-Aldrich GmbH, Steinheim is the correct city nameSteinhiem, Germany). Ferulic acid (97%) and chlorogenic acid (95%) were purchased from the European Pharmacopoeia (EP).

### 2.2. Vegetal Materials and Preparation of the Extracts

Blackcurrant plants (fruits and leaves) were harvested from spontaneous flora from the hilly area of OLT county (44°26′00″ N, 24°22′00″ E), Romania, in August 2022. The pomace was defined as the solid remains after mechanical pressing of juice extraction.

For antioxidant determinations and inhibition of free radical and lipid peroxidation, the air-dried vegetal materials were finely ground with a Grindomix GM 200 mill (Retsch, Germany) and macerated with methanol (80%) for 24 h at room temperature without light. A 1500 g for 10 min centrifugation step was applied after extraction, and the supernatant was collected for further analysis. At the beginning of the study, proximate composition was determined (Delete this phrase), and results regarding dry matter content exceeded 93% for all samples considered for the study.

For individual polyphenol identification and quantification, the extraction solvent consists of water/methanol/acetic acid in a ratio of 69:30:1, *v*/*v*/*v*, and the ratio used in the study was 0.5 g dried sample/10 mL solvent. Sixty minutes of incubation in a shaking water bath (Memmert, Schwabach, Germany) at 50 °C, 4000 rpm centrifugation for 15 min and filtration were the following steps for polyphenol extracts.

### 2.3. Determination of Individual Polyphenolic Compounds

The analytical method used for the identification and quantification of polyphenolic compounds was previously described by [14]. Vanquish Core HPLC with a DAD system (Thermo Fisher Scientific, Bremen, Germany) was used to determine polyphenols. The analytical column was BDS HyperSil C18 (250 × 4 mm and 5 µm particle size). Acetic acid (1%) (solvent A), methanol (solvent B), and acetonitrile (solvent C) were the components of the gradient, and the elution program was: 0–15 min—5% (B), 5% (C); 15–20 min—4% (B), 15% (C); 20–25 min—3% (B), 25% (C); 25–40 min—2% (B), 38% (C); 40–50 min—5% (B), 5% (C). The optimum flow rate was established at 0.5 mL/min. Individual standards were used for the identification of phenolic compounds.

### 2.4. In Vitro Simulated Gastrointestinal Digestion of Polyphenols

Static digestion, the INFOGEST 2.0 protocol, was applied for in vitro simulation of gastrointestinal phases of digestion [9].

Simulated salivary fluid (SSF) was prepared, consisting of 15.1 mM KCl, 3.7 mM KH_2_PO_4_, 13.6 mM NaHCO_3_, 0.15 mM MgCl_2_ (H_2_O)_6_, 0.06 mM (NH_4_)_2_CO_3_, and 1.5 mM CaCl_2_.

Simulated gastric fluid (SGF) was prepared, consisting of 6.9 mM KCl, 0.9 mM KH_2_PO_4_, 25 mM NaHCO_3_, 47.2 mM NaCl, 0.10 mM MgCl_2_ (H_2_O)_6_, 0.50 mM (NH_4_)_2_CO_3_, and 0.15 mM CaCl_2_.

Simulated intestinal fluid (SIF) was prepared, consisting of 6.8 mM KCl, 0.8 mM KH_2_PO_4_, 85 mM NaHCO_3_, 38.4 mM NaCl, 0.33 mM MgCl_2_ (H_2_O)_6_, and 0.6 mM CaCl_2_.

Simulated oral digestion phase: A total of 5 g of vegetal material were mixed with 3.5 mL SSF and gently homogenized. A quantity of 0.5 mL amylase solution prepared in SSF (1500 U/mL), 25 µL CaCl_2_ (0.3 M), and 975 µL of distilled water was added to the mixture. The obtained solution was incubated at 37 °C for 2 min under constant shaking without light.

Simulated gastric digestion phase: The bolus resulting from the previous phase was mixed with 7.5 mL SGF and gently homogenized. A quantity of 1.6 mL of pepsin solution prepared in SGF (250 U/mg), 5 µL CaCl_2_ (0.3 M), and 695 µL of distilled water were added to the mixture. The pH was adjusted to 3.0 using HCl (1M). The obtained solution was incubated at 37 °C for 2 h under constant shaking without light.

Simulated intestinal digestion phase: The chyme resulting from the previous phase was mixed with 11 mL SIF and homogenized. A quantity of 5 mL pancreatin solution prepared in SIF, 2.5 mL bile extract (160 mM), 40 µL CaCl_2_ (0.3 M), and 1.31 mL of distilled water were added to the mixture. The pH was adjusted to 7.0 using NaOH (1M). The obtained solution was incubated at 37 °C for 2 h under constant shaking without light.

At the end of every digestion phase, the mixtures were centrifuged at 4500 rpm, 4 °C, and 15 min and the supernatants obtained were used for polyphenol determination and recovery index calculation.

The recovery index is an arithmetic parameter calculated according to the [15] formula, and it evaluates the effect of studied vegetal materials on the digestion of some phenolic classes.

RI (%) = B/A × 100

where A represents the polyphenol concentration in vegetal material, and B represents the polyphenol released during simulated digestive steps.

### 2.5. Antioxidant Activity Assays

Four different analytical methods were used for the evaluation of the antioxidant potential of the vegetal materials considered for this study.

DPPH, ABTS, and phosphomolybdenum method assays were performed according to the details described in [16]. A DPPH solution prepared in methanol (0.2 mM) is mixed with sample extract and distilled water (2:0.4:1.6; *v*/*v*/*v*), and the absorbance was recorded using a spectrophotometer (Jasco V-530, Japan Servo Co., Ltd., Tokyo, Japan) at 517 nm. ABTS solution (7 mM ABTS and 2.4 mM potassium persulfate) was prepared and left to react for 12 h in the dark. The absorption of the solution was adjusted to 0.7. The ABTS solution was mixed with sample extract (6:0.04, *v*/*v*), and the absorbance was recorded at 734 nm. Standard calibration curves using Trolox as a reference were used to determine DPPH and ABTS concentrations. The results were reported as mmol eq trolox/kg sample.

The phosphomolybdenum method was applied by reading at 695 nm the absorbance of a mixture containing the sample extract (0.2 mL) and 4 mL of reagent solution (0.6 M sulfuric acid, 28 mM sodium phosphate, and 4 mM ammonium molybdate). Ascorbic acid was used as the reference standard. The reagent solution (4 mL) mixed with 0.2 mL of ethanol was used as a blank, and the results were expressed as mmol eq ascorbic acid/kg sample.

#### 2.5.1. Radical Scavenging Assays

The methods used for radical scavenging potential were previously described elsewhere [17].

Superoxide anion inhibition evaluation: different concentrations of samples were mixed with solutions of NBT 50 mM (1 mL) and NADH 78 mM (1 mL), and the reaction was started with 1 mL PMS 10 mM. Absorbances were read at 560 nm. Inhibition was calculated by subtracting the control from the sample value and dividing it by the control value.

Hydroxyl anion inhibition evaluation: different concentrations of samples were mixed with a reaction solution containing 200 μL KH_2_PO_4_–KOH (100 mM), 200 μL deoxyribose (15 mM), 200 μL FeCl_3_ (500 μM), 100 μL EDTA (1 mM), and 100 μL ascorbic acid (1 mM), 100 μL H_2_O_2_ (10 mM), and one hour at 370 C was the first incubation step. TBA (1%) and TCA (2.8%) were added to the mixture, 1 mL each, followed by the second incubation step at 80 °C for 20 min. Absorbances were read at 532 nm. Inhibition was obtained by subtracting the absorbance of the control from the sample and dividing that by the control absorbance.

#### 2.5.2. In Vitro Induced Lipid Peroxidation

For inducing lipid peroxidation using an oxidation system consisting of FeCl_2_ (100 µM) and ascorbic acid (500 µM), the method described by [17] was applied. Fresh chicken meat samples were homogenized with ice-cold Tris HCl buffer, 0.15 M, pH 7.4 (1:9, *v*/*v*). A centrifugation step was applied (14,000 g for 20 min), followed by supernatant collection. The methanolic extract (1000 ppm) was added to the meat sample extract, and the peroxidation was induced by adding the oxidation mixture (FeCl_2_/ascorbic acid system). The mixture was incubated at 37 °C for an hour. The TBARS method (mixture of sample solution and TCA (7.5%) and BHT (0.8%) react with TBA solution (0.8%) for 50 min at 80 °C) was applied for quantification of malondialdehyde in meat samples using derivative spectrometry. The standard curve was obtained by plotting different concentrations of 1,1,3,3-tetra methoxy propane (TMP) and the respective absorbance recorded.

### 2.6. Statistical Analysis

The results declared in the tables or figures represent the average concentrations of nutrients determined individually in triplicate. The data obtained were statistically interpreted using analysis of variance (ANOVA), and the significance was set at *p* < 0.05, as assessed by Tuckey’s test. The XLSTAT software (v.19.01, Addinsoft, Paris, France) was used for statistical interpretation and correspondence analysis. Prism GraphPad software v. 9.03 (San Diego, CA, USA) was used to represent the inhibition of lipid peroxidation.

## 3. Results

### 3.1. Effect of Simulated In Vitro Digestion on Bioactive Composition of Blackcurrant

The phenolic composition of fruits, leaves, and pomace is presented in Table 1. The data presented revealed important differences between polyphenol concentrations in blackcurrant extracts, from which the leaves are the main source of phenolic acids, or flavonoids. No significant differences in phenolic acid content were noticed between the pomace and fruit samples. Pomace is an important source of hydroxybenzoic acid, while the fruits are rich in hydroxycinnamic acids, especially chlorogenic acid. The identified flavonoids presented almost similar concentrations, except for catechin, which is a flavanol dominant in pomace samples.

Before digestion, leaf extract presented the highest content of phenolics and flavonoids, followed by pomace and fruits, as presented in Table 1. Changes in the polyphenol profile were observed after the digestion process, depending on the sample type and digestion phase. A similar pattern was noticed between fruits and pomace, where the concentrations in the oral and gastric phases were lower but increased in the intestinal ones. However, the acquired results were smaller when compared with undigested samples. However, the leaves maintained, after digestion, the highest content of phenolic acids and flavonoids. The results obtained after every digestion step of fruits, pomace, and leaves are presented in Table 2, Table 3 and Table 4.

After every simulated digestion step, the concentrations of polyphenols determined in leaves increased even tenfold (intestinal phase) compared with the other two types of samples. The results obtained are not unexpected, taking into account the polyphenol profile determined before digestion. The other two samples (fruits and pomace) presented comparable results, with hydroxycinnamic acids being more abundant in fruit extracts, while hydroxybenzoic acids and flavanols were present in high quantities in pomace.

The content of phenolic acids decreased after the intestinal digestion phase compared to undigested samples, with a recovery index between 52 and 73% for pomace, leaves, and fruits, respectively. Flavonoids presented good stability during the in vitro digestion conditions in fruit and pomace extracts (77–93%), while the degrading process led to a lower recovery index in leaf extracts (52–56%). Regarding phenolic acids, gallic acid presented higher stability in all studied extracts. A different trend was observed for coumaric acid, which registered the lowest recovery index from the phenolic acid class for all types of blackcurrant extracts. The flavonoid class was subdivided into flavanols and flavonol groups. From the flavanols group, the determined monomers catechin and epicatechin presented higher stability after intestinal digestion in fruits and pomace samples, but degradation processes were noticed in leaf extracts. The same trend was observed for flavonols, with rutin concentrations being affected after the digestion process in leaf samples.

### 3.2. The Blackcurrant Extracts (Fruits, Pomace and Leaves) Antioxidant Capacity

The antioxidant capacity of three different sample extracts belonging to blackcurrant was evaluated by applying four different analytical methods: phosphomolybdate assays, DPPH and TEAC assays, and iron chelation capacity. The results obtained are presented in Table 5.

The analytical data from Table 5 showed that all four methods revealed that the maximum antioxidant potential was exhibited by leaf samples. Regarding the other two extracts (fruits and pomace), the phosphomolybdate assay indicated that pomace extracts possess a higher antioxidant activity of hydrophilic compounds evaluated as equivalents of ascorbic acid, while fruits are more powerful than pomace regarding the scavenging abilities of DPPH and ABTS radicals. The iron chelation properties respect the following order: leaves > pomace > fruits.

### 3.3. The Effect of Blackcurrant Extracts (Fruits, Pomace and Leaves) on Inhibition of Free Radicals

**Figure 1:**
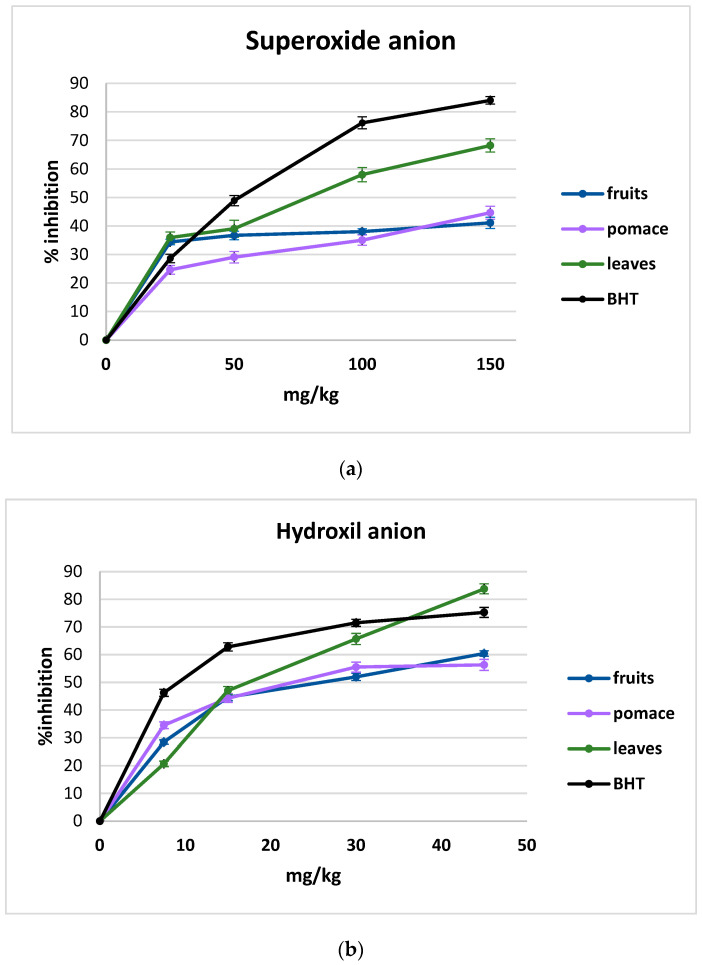
Superoxide anion (**a**) and hydroxyl anion (**b**) scavenging activities of fruits, pomace, and leaves of blackcurrant were compared with standard BHT. The data represent the percentage of radicals’ inhibition.

The free radical scavenging activities of blackcurrant extracts were evaluated using hydroxyl anion and superoxide anion. Different concentrations of extracts were exposed to radicals, and a decrease in radical absorbance was recorded. The obtained results are graphically represented in Figure 1a,b.

The data presented in Figure 1a,b showed that BHT is the most effective scavenger of superoxide and hydroxyl radicals. Considering the three blackcurrant samples, increasing concentrations of plants exhibited increasing inhibition power of free radicals. The analytical evaluation of 100 and 150 mg/kg of plant extract (for superoxide anion inhibition) and 30–45 mg/kg plant extract (for hydroxyl radical anion inhibition) revealed no significant differences between pomace and fruit behavior. However, the leaf samples exhibited over 50% higher superoxide anion when compared with fruits and pomace and an average of 30% higher hydroxyl anion.

### 3.4. The Effect of Blackcurrant Extracts (Fruits, Pomace, and Leaves) on Inhibition of Lipid Peroxidation

Iron-induced lipid peroxidation was applied to study the potential of blackcurrant fruits, pomace, and leaves to counteract the oxidation process that occurs in lipid matrices. In our study, fresh chicken breast was treated with a solution containing trivalent iron and ascorbic acid as an oxidation system, and the final peroxidation products were recorded as malondialdehyde (MDA) concentrations. Table 6 presents the influence of the oxidation system on the lipid matrix of meat and the influence of different extracts (BHT and blackcurrant extracts) on delaying the process. The data showed no statistical differences between the non-peroxidized sample and meat oxidized with a BHT inhibitory solution, proving that BHT is a good choice for an antioxidant standard. The leaf extract registered MDA values relatively close to BHT. The fruit and pomace samples presented higher values but were significantly reduced compared to oxidized fresh meat.

**Figure 2:**
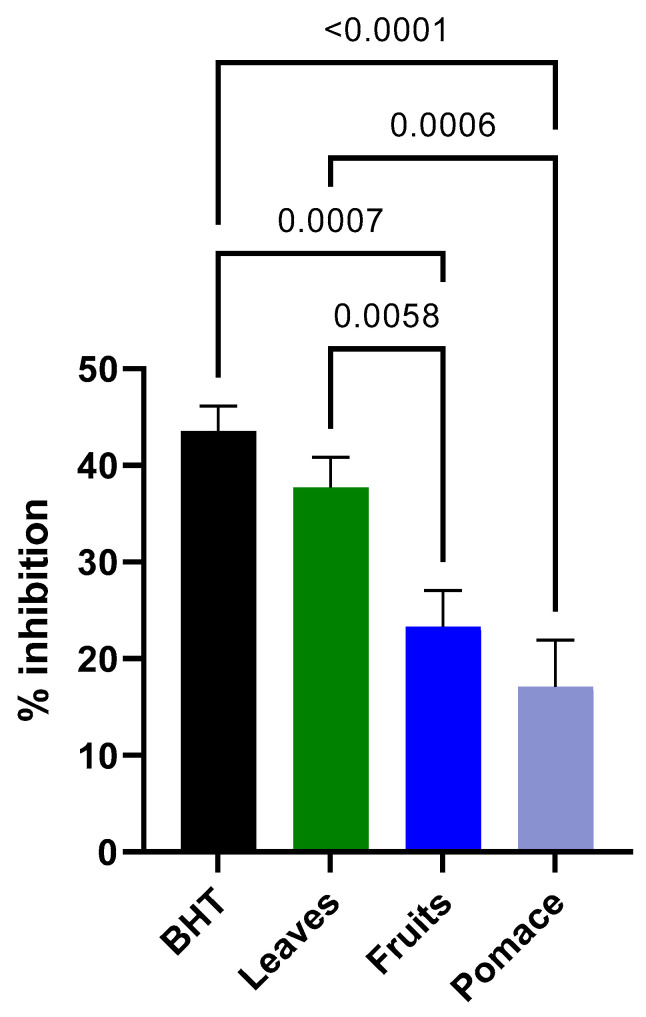
Effect of blackcurrant extracts on inhibition of lipid peroxidation.

In Figure 2, the inhibition potential evaluation of extracts showed there are no statistical differences between BHT and leaf extract-treated samples. Fruits and pomace-treated samples exhibited significantly less inhibition power compared to the rest of the samples.

**Figure 3:**
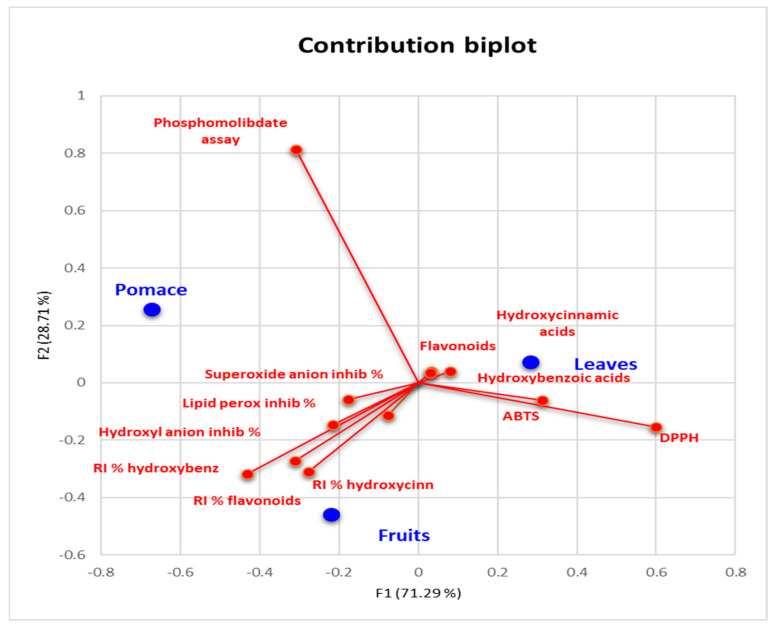
Biplot contribution of the studied analytical parameters in the vegetal materials space.

The relationship between analyzed parameters and vegetal materials was studied by applying a statistical tool called correspondence analysis, and the results are displayed in Figure 3.

The first observation regarding the correspondence analysis of the described system is related to the independence test. The results indicated that the chi-square observed value > the chi-square critical value and *p* < 0.0001, which means that our studied parameters are significantly associated with the vegetal materials. The larger distance to the origin indicates dissimilarities between parameters and the mean profile. In our study, the inhibition potential against biological radicals, lipid peroxidation, and concentrations of polyphenol classes present the shortest distance to the origin, indicating that those parameters are closely related to the mean of the system. According to the squared cosine table, all studied parameters are attributed to factor F1, except the phosphomolybdenum assay, and regarding vegetal materials, leaves and pomace are related to factor F1 and fruits to factor F2. It can be observed that polyphenol classes and non-biological radicals (ABTS and DPPH) are plotted on the opposite side with recovery indices, biological radicals, and lipid peroxidation inhibition. This graphical arrangement suggests that the parameters are clusters with different profiles. Leaves and pomace are also on opposite sides of the F1 axis, indicating different profiles. Correspondence analysis indicated the association of leaves with polyphenol classes, while fruits were associated with their recovery indices.

## 4. Discussion

Blackcurrant fruits exist in the traditional medicine of many European countries and are known for their therapeutic effects, including anti-inflammatory and immunomodulatory effects, as well as their antioxidant properties, with anthocyanins being the most studied polyphenol class [1]. The current study evaluated the phenolic profile of blackcurrant extracts (fruits, pomace, and leaves) before and after a simulated in vitro model of human digestion. Considering the selected phenolic compounds for this study, the results obtained revealed that the leaves are the most important source of polyphenols in undigested samples. Different authors investigated the phenolic constituents of some plants belonging to the “berry” family (cranberry, chokeberry, bilberry, and blackcurrant). They observed no correlation between the polyphenol compounds from fruits and leaves, and they found that phenolic acids and flavonols are more abundant in leaves than in fruits [5]. Berries are colored fruits, with anthocyanins being the major contributor to the polyphenolic profile [13]. Anthocyanins are water-soluble pigments within the flavonoid family, comprising hundreds of species, with the top six most abundant representing 90% of the total. Several factors like pH, temperature, humidity, or oxygen levels influence their stability, and the bioavailability of anthocyanins is presumed to be less than 1% [18]. Other authors postulated that high-molecular-weight phenolic compounds, such as anthocyanins and tannins, show low bioavailability, while their metabolites (low-molecular-weight polyphenols) such as phenolic acids were determined in tissues, and no direct relationship was found between their concentrations in organisms and undigested plant samples [2]. Human nutrition studies regarding the metabolism of polyphenols concluded that the major metabolites of anthocyanins identified in the circulation post-consumption are phenolic acids like vanillic acid, syringic acid, caffeic acid, and ferulic acid, among others [19,20,21].

In our study, six major phenolic acids, corresponding to gallic, vanillic, hydroxybenzoic, ferulic, chlorogenic, and methoxy-cinnamic acids, and two major flavonoids (epigallocatechin and epicatechin), were determined in digested fractions of leaves. A similar range of values was recorded in fruits only for chlorogenic acid from the phenolic acid class and epicatechin from the flavonoid class. Gallic and hydroxybenzoic compounds from the phenolic acid group and flavonoids such as catechin and epicatechin were the major compounds determined in digested fractions of pomace.

The RI calculated for phenolics determined in digested leaves showed that gallic acid is the most bioaccessible compound, followed by vanillic, ferulic, and cinnamic acids. The polyphenols determined in the digested fruit samples proved to be more bioaccessible than polyphenols from leaves, gallic, metoxicinnamic, chlorogenic acids, and flavonoids like epicatechin and catechin, which recorded the highest values of RI. The same pattern was observed for pomace, except for chlorogenic acid, which was not detected in the samples.

The most bioaccessible phenolic acid was proven to be gallic acid (Table 2, Table 3 and Table 4), determined in all three morphological parts of blackcurrant (RI between 93.43 and 109.49%). Gallic acid is released from the digestive matrix by mechanical action and enzymatic activity, including hydrolysis, depolymerization, and deglycosylation. Other authors observed an increment in gallic acid concentrations after digestive simulated steps, and they attributed this fact to the hydrolysis of galloylated molecules as hydrolyzable tannins [22,23]. Another theory presumed that the increase in phenolic acids and flavonols could be attributed to the release of polyphenol molecules from proteins and other biomolecules [24]. Vanillic acid is another phenolic acid from the hydroxybenzoic acid class and is a product of anthocyanin hydrolysis via protocatechuic acid metabolism [2]. This mechanism can explain the decrease in protocatechuic acid concentrations and the increase in vanillic acid during the digestion process, mainly observed in the leaf’s samples, while in fruit and pomace samples, the protocatechuic acid was not detectable.

Hydroxycinnamic acids represent almost 70% of the phenolic acids in fruits and leaves, and the main components are chlorogenic and ferulic acids. Chlorogenic acid is a phenolic acid derived from caffeic acid and a metabolite of acylated anthocyanins through enzymatic deglycosylation and spontaneous cleavage. Caffeic acid is another compound from the hydroxycinnamic acid class, a product of protocatechuic acid cleavage, and vanillic acid isomers are its metabolites [2]. The main stability during the digestion process was detected in leaf samples, and in all three types of vegetal samples, the bioaccessibility indices were greater than protocatechuic acid. Ferulic acid is a product of some processes like anthocyanin catabolism, spontaneous cleavage of protocatechuic acid, or spontaneous carboxylation of caffeic acid. The concentrations recovered during the digestion process registered important values, which led to recovery indices with higher percentages in fruit and leaf samples.

The results presented in Table 2, Table 3 and Table 4 showed important concentrations of flavonoids for the analyzed vegetal samples. These observations can be explained by the good recovery of this phenolic class during the digestion step or by the biotransformation process of other polyphenols with high molecular weight. Flavonoids like flavan-3-ols are monomeric forms, while proanthocyanidins are polymers or oligomers of the same chemical forms, and their digestibility is related to the polymerization degree [25]. Some authors considered that a mildly acidic environment favors the depolymerization process of proanthocyanidins, which can explain the increased concentrations of flavanols in gastrointestinal phases. The same authors considered that, under pancreatic enzymes’ influence, they are degraded, resulting in phenolic acids as final products [2]. For example, gallic acid is a phenolic compound that is unstable in an alkaline environment, but it can also be a final metabolite of proanthocyanidins, gallotannins, and ellagitannins.

### 4.1. Blackcurrant Extracts (Fruits, Pomace, and Leaves): Antioxidant Capacity and Inhibition of Biological Free Radicals

The polyphenols existing in plants possess the antioxidant capacity for their defense, but they also present these properties, making them essential in animal and human nutrition. Antiradical activities, chelating properties, and the Folin Ciocalteu method are the most popular determinations that describe the total antioxidant activity of polyphenols [26]. The antioxidant assays are based on the same chemical principle: a colored radical is generated (green ABTS and purple DPPH) or a redox-active compound, and the radical scavenging activity of the studied sample or the reducing capacity is quantified using an established relevant standard (trolox, gallic acid, vitamin C, etc.) [27]. The methods described before using non-physiological radicals. Other antioxidant assays use biological radicals (O2•−, •OH, •NO) to determine the scavenging abilities of plant extracts [28].

In the current study, a multimethod approach was applied for the evaluation of antioxidant capacity, which describes the morphological parts of blackcurrant. The antioxidant capacity and the health-promoting effects of berries are closely related to the presence of anthocyanins in their chemical structure; they are also responsible for the color of fruits [29]. Other authors attribute the antioxidant power of blackcurrant fruits to their cyanidin and delphinidin contents [30]. Ref. [31] reported that the leaves of currant plants exhibit stronger anti-inflammatory activity due to their increased antioxidant capacity compared with fruits. The same authors expressed the antioxidant activity using the ABTS, DPPH, and FRAP methods and found that the strongest properties were related to the DPPH radical. In our study, the leaf extracts consistently yielded heightened results across all antioxidant methods employed, showing their superior antioxidant potential compared to other extract types.

Iron plays an important role in free radical generation, participating in Fenton reactions with hydroxyl radicals’ formation as final products or Haber–Weiss Cycle reactions with superoxide anions formation. The iron-chelating properties of polyphenols are attributed to the presence of an orthodihydroxy group in the chemical structure of polyphenols. The iron binding capacity of phenolic acids was studied by [32], and they concluded that hydroxycinnamic acids (caffeic acid and chlorogenic acid) presented a higher binding capacity than hydroxybenzoic acids (gallic acid and protocatechuic acid). The chelation of a greater capacity of hydroxycinnamic acids can be attributed to the ethylene group from the benzene ring and carboxylic group [32]. In our study, the phenolic acids were determined in all three blackcurrant samples, from which the leaves proved to be the most abundant source. The concentrations of hydroxycinnamic acids in leaves are more than double compared with hydroxybenzoic acids, with ferulic and chlorogenic acids being the major compounds. According to this phenolic structure, the iron binding capacity determined indicated the leaves as the best ligands for iron.

The results regarding the scavenging abilities of ethanolic extracts on biological radicals were presented in the current study. Superoxide and hydroxyl radicals were exhibited in a dose-dependent manner, and increasing concentrations of extracts produced an increased inhibition effect for all radicals studied. Superoxide anion and hydroxyl radical are considered to be initiators of lipid peroxidation, proving a positive correlation between this radical and fat oxidation products [33].

For both studied free radicals, at the maximum extracts’ concentrations, the inhibition power respects the following order: leaves > pomace > fruits. The value of IC50 (the plant concentration that exhibits a 50% inhibitory effect) respects the inverse trend presented before. The plant extracts scavenging activities were compared with synthetic antioxidant activity (BHT) using the same concentrations for the study. For superoxide anions, the calculated IC50 values were 222.84 mg/L (R2 = 0.9522) for blackcurrant fruits, 121.74 mg/L (R2 = 0.9879) for pomace, and 80.12 mg/L (R2 = 0.9779) for leaves. The synthetic antioxidant used as a reference (BHT) registered IC_50_ = 59.87 mg/L (R2 = 0.9228). In the case of hydroxyl radicals, the same type of analytical interpretation provided the following results: 29.06 mg/L (R2 = 0.8928) for fruits, 28.44 mg/L (R2 = 0.8523) for pomace, and 21.65 mg/L (R2 = 0.9445) for leaves, and for BHT, the calculated IC_50_ was 22.95 mg/L (R2 = 0.8257). The results obtained showed that the leaf extract was the most effective morphological part of blackcurrant in scavenging the free radical’s activity. A study published in 2018 [34] revealed that extracts obtained from leaves are more powerful in trapping free radicals than berry extracts, studying 24 different plants from the berry family. The same authors found that non-flavonoid phenolics (phenolic acids and other phenolics) were significantly correlated with radical scavenging activity. This observation can sustain our results, proving (Table 1) that the leaves are the main source of phenolic acids.

### 4.2. The Effect of Blackcurrant Extracts on Lipid Peroxidation Inhibition

The inhibitory effect of plant extracts on lipid peroxidation was proven by in vitro studies focused on berry fruits or leaves. The authors in [35] studied the effect of different berry fruit anthocyanin-rich extracts (blackberry, blueberry, strawberry, and chokeberry) on in vitro oleic acid-induced hepatic steatosis and concluded that blackberry registered the highest inhibitory effect. Another study on Rose Myrtle berries revealed a remarkable effect of flavonoids extracted on the inhibition of lipid peroxidation, using fresh lard as a substrate [36]. Blackcurrant ethanolic extract was used for the evaluation of anthocyanins’ ability to counteract lipid and protein oxidation, proving their efficacy in a pork patties lipid matrix [37]. Bilberry, cranberry, and raspberry leaves were comparatively studied for their effect on in vitro-induced lipid peroxidation of meat and showed that bilberry leaves possess the strongest inhibitory effect [38].

Our study used chicken breast as a lipid matrix, and the peroxidation process was induced by the Fe^3+^/ascorbic acid system. The comparison between three blackcurrant extracts showed that the leaves displayed a remarkable effect on delaying the oxidation process without significant differences compared with a synthetic antioxidant (BHT). The fruits and pomace extracts presented similar behavior and an average of 20% inhibition effect. Considering the polyphenolic profile presented in the current study, we can attribute the evaluated effect on phenolic acids and flavonoids determined in quantities over 18-fold greater in the leaf samples than in fruit or pomace. A possible reaction mechanism connects the radical scavenging activity (by donating electrons or hydrogen to free radicals) to inhibit lipid oxidation by blocking the radical chain reaction [37].

## 5. Conclusions

This study presented the effects of in vitro gastrointestinal digestion conditions on the polyphenol profile and antioxidant potential of blackcurrant fruits, pomace, and leaves. The results obtained were related to the sample type. The recovery index presented higher values in the case of fruit extracts, indicating higher bioaccessibility. Flavonoids are the phenolic group with high stability for pomace and some individual phenolics like gallic, vanillic, ferulic, or cinnamic acids for leaves. The antioxidant evaluation and inhibition of biological and non-biological radicals indicate the leaf extract is the most powerful antioxidant studied. The results obtained proved that not only fruits but also the blackcurrant byproducts (pomace and leaves) are a promising source of bioaccessible antioxidants with potential benefits in animal nutrition.

## Figures and Tables

**Table 1:** The phenolic profile of blackcurrant extracts (fruits, leaves, and pomace).

	Fruits mg/g	Leaves mg/g	Pomace mg/g	SEM	*p*-Value
**Phenolic acids**	**0.628 ^b^**	**11.39 ^a^**	**0.612 ^b^**	**0.443**	0.0001
***Hydroxybenzoic acids***	***0.185* ^b^**	***3.319* ^a^**	***0.489* ^b^**	***0.183***	0.0001
Gallic acid	0.048 ^c^	0.506 ^a^	0.113 ^b^	0.004	0.0001
Vanillic acid	0.034 ^b^	1.166 ^a^	0.035 ^b^	0.016	0.0001
Syringic acid	0.030 ^b^	0.290 ^a^	0.000 ^b^	0.010	0.0001
Hydroxybenzoic acid	0.050 ^b^	1.153 ^a^	0.300 ^b^	0.176	0.010
Ellagic acid	0.023 ^b^	0.189 ^a^	0.041 ^b^	0.010	0.0001
Protocatechuic acid	0.000 ^b^	0.014 ^a^	0.000 ^b^	0.000	0.0001
					
***Hydroxycinnamic acids***	***0.443* ^b^**	***8.074* ^a^**	***0.123* ^b^**	***0.261***	0.0001
Chlorogenic acid	0.316 ^b^	2.293 ^a^	0.000 ^c^	0.038	0.0001
Caffeic acid	0.021 ^b^	0.176 ^a^	0.023 ^b^	0.017	0.001
Metoxicinnamic acid	0.013 ^b^	1.078 ^a^	0.039 ^b^	0.022	0.0001
Ferulic acid	0.087 ^b^	3.588 ^a^	0.050 ^b^	0.211	0.0001
Coumaric acid	0.006 ^b^	0.925 ^a^	0.011 ^b^	0.016	0.0001
Cinnamic acid	nd ^1^	0.014 ^a^	nd ^1^	0.000	0.0001
					
**Flavonoids**	**0.183 ^c^**	**2.809 ^a^**	**0.411 ^b^**	**0.026**	0.0001
***Flavanols***	***0.18 ^c^***	***2.62* ^a^**	***0.40* ^b^**	***0.028***	0.0001
Epigallocatechin	nd ^1^	1.369	nd ^1^	-	-
Catechin	0.031 ^b^	0.088 ^b^	0.253 ^a^	0.018	0.0001
Epicatechin	0.146 ^b^	1.163 ^a^	0.147 ^b^	0.053	0.0001
					
***Flavonols***	***0.006* ^b^**	***0.189* ^a^**	***0.012* ^b^**	***0.011***	0.0001
Rutin	0.006 ^b^	0.184 ^a^	0.010 ^b^	0.010	0.0001
Quercetin	nd ^1^	0.005 ^a^	0.002 ^b^	0.001	0.0001
					
**Stilbene**	***nd* ^1^**	***0.006***	***nd* ^1^**	*-*	-
Resveratrol	nd ^1^	0.006	nd ^1^	-	-

^1^ nd—not detected; *p*-value represents the level of significance, and different letters within each row mean significant differences between samples.

**Table 2:** Polyphenol concentrations in blackcurrant fruits after digestion steps and their recovery index.

Phenolic Compounds in Fruit Samples	Digestive Phases					
	Oral	Gastric	Intestinal	SEM	*p*-Value	RI ^1^
	mg/g	mg/g	mg/g			%
**Phenolic acids**	**0.220 ^b^**	**0.262 ^b^**	**0.489 ^a^**	**0.026**	**0.0001**	**72.81**
***Hydroxybenzoic acids***	***0.064* ^c^**	***0.080* ^b^**	***0.140* ^a^**	***0.001***	**0.0001**	***73.94***
Gallic acid	0.018 ^c^	0.021 ^b^	0.045 ^a^	0.000	0.0001	93.43
Vanillic acid	0.014 ^b^	0.016 ^b^	0.026 ^a^	0.000	0.0001	76.50
Syringic acid	0.011 ^b^	0.013 ^b^	0.021 ^a^	0.000	0.0001	68.85
Hydroxybenzoic acid	0.014 ^c^	0.018 ^b^	0.033 ^a^	0.000	0.0001	66.68
Ellagic acid	0.008 ^c^	0.011 ^b^	0.014 ^a^	0.000	0.0001	64.26
Protocatechuic acid	nd ^2^	nd ^2^	nd ^2^			nd ^2^
						
***Hydroxycinnamic acids***	***0.156* ^c^**	***0.182* ^b^**	***0.348* ^a^**	***0.027***	**0.0001**	***71.68***
Chlorogenic acid	0.103 ^c^	0.134 ^b^	0.255 ^a^	0.001	0.0001	80.80
Caffeic acid	0.005 ^b^	0.006 ^b^	0.012 ^a^	0.000	0.0001	57.75
Metoxicinnamic acid	0.002 ^c^	0.007 ^b^	0.011 ^a^	0.000	0.0001	89.86
Ferulic acid	0.042 ^b^	0.033 ^c^	0.067 ^a^	0.001	0.0001	76.68
Coumaric acid	0.003 ^a^	0.002 ^b^	0.003 ^a^	0.000	0.0001	53.29
Cinnamic acid	nd ^2^	nd ^2^	nd ^2^			nd ^2^
						
**Flavonoids**	**0.183 ^a^**	**0.079 ^c^**	**0.095 ^b^**	**0.014**	**0.0001**	**91.04**
***Flavanols***	***0.08* ^b^**	***0.09* ^b^**	***0.17* ^a^**	***0.001***	**0.0001**	***89.88***
Epigallocatechin	nd ^2^	nd ^2^	nd ^2^			nd ^2^
Catechin	0.013 ^b^	0.012 ^b^	0.026 ^a^	0.001	0.0001	82.60
Epicatechin	0.064 ^c^	0.080 ^b^	0.142 ^a^	0.002	0.0001	97.15
						
***Flavonols***	***0.001* ^c^**	***0.003* ^b^**	***0.005* ^a^**	0.000	**0.0001**	***93.36***
Rutin	0.001 ^c^	0.003 ^b^	0.005 ^a^	0.000	0.0001	93.36
Quercetin	nd ^2^	nd ^2^	nd ^2^			nd ^2^
						
**Stilbene**						
Resveratrol	***nd* ^2^**	***nd* ^2^**	***nd* ^2^**			***nd* ^2^**

^1^ RI—recovery index, ^2^ nd—not detected. *p*-value represents the level of significance, and different letters within each row mean significant differences between samples.

**Table 3:** Polyphenol concentrations in blackcurrant pomace after digestion steps and their recovery index.

Phenolic Compounds in Pomace Samples	Digestive Phases					
	Oral	Gastric	Intestinal	SEM	*p*-Value	RI ^1^
	mg/g	mg/g	mg/g			%
**Phenolic acids**	**0.237 ^b^**	**0.231 ^b^**	**0.383 ^a^**	**0.024**	**0.0001**	**56.87**
***Hydroxybenzoic acids***	***0.188* ^b^**	***0.193* ^b^**	***0.314* ^a^**	***0.009***	**0.0001**	***61.08***
Gallic acid	0.036 ^b^	0.040 ^b^	0.123 ^a^	0.001	0.0001	109.09
Vanillic acid	0.015 ^b^	0.014 ^b^	0.017 ^a^	0.001	0.0023	48.47
Syringic acid	nd ^2^	nd ^2^	nd ^2^	0.000	0.0001	nd ^2^
Hydroxybenzoic acid	0.106 ^c^	0.131 ^b^	0.160 ^a^	0.007	0.0001	53.22
Ellagic acid	0.031 ^a^	0.008 ^c^	0.014 ^b^	0.000	0.0001	33.54
Protocatechuic acid	nd ^2^	nd ^2^	nd ^2^			nd ^2^
						
***Hydroxycinnamic acids***	***0.049* ^b^**	***0.037 ^c^***	***0.069* ^a^**	***0.027***	**0.0001**	***52.65***
Chlorogenic acid	nd ^2^	nd ^2^	nd ^2^			nd ^2^
Caffeic acid	0.011 ^a^	0.009 ^b^	0.012 ^a^	0.000	0.0010	51.77
Metoxicinnamic acid	0.018 ^b^	0.021 ^b^	0.032 ^a^	0.001	0.0001	82.84
Ferulic acid	0.015 ^b^	0.005 ^c^	0.022 ^a^	0.001	0.0001	43.14
Coumaric acid	0.004 ^a^	0.002 ^b^	0.004 ^a^	0.000	0.0001	32.86
Cinnamic acid	nd ^2^	nd ^2^	nd ^2^			nd ^2^
						
**Flavonoids**	**0.318 ^b^**	**0.314 ^b^**	**0.364 ^a^**	**0.014**	**0.0010**	**80.75**
***Flavanols***	***0.312* ^b^**	***0.309* ^b^**	***0.356* ^a^**	***0.014***	**0.0010**	***83.68***
Epigallocatechin	nd ^2^	nd ^2^	nd ^2^			nd ^2^
Catechin	0.192 ^b^	0.197 ^b^	0.216 ^a^	0.008	0.0001	85.31
Epicatechin	0.119 ^b^	0.113 ^b^	0.140 ^a^	0.008	0.0001	82.04
						
***Flavonols***	***0.006* ^b^**	***0.005* ^b^**	***0.009* ^a^**	0.000	**0.0001**	*77.82*
Rutin	0.005 ^b^	0.004 ^b^	0.007 ^a^	0.000	0.0001	72.81
Quercetin	0.001 ^b^	0.001 ^b^	0.002 ^a^	0.000	0.0001	82.84
						
**Stilbene**						
Resveratrol	***nd* ^2^**	***nd* ^2^**	***nd* ^2^**			***nd* ^2^**

^1^ RI—recovery index, ^2^ nd—not detected. *p*-value represents the level of significance, and different letters within each row mean significant differences between samples.

**Table 4:** Polyphenol concentrations in blackcurrant leaves after digestion steps and their recovery index.

Phenolic Compounds in Leaf Samples	Digestive Phases					
	Oral	Gastric	Intestinal	SEM	*p*-Value	RI ^1^
	mg/g	mg/g	mg/g			%
**Phenolic acids**	**1.372 ^c^**	**3.666 ^b^**	**6.255 ^a^**	**0.132**	**0.0001**	**62.96**
***Hydroxybenzoic acids***	***0.297 ^c^***	***0.906* ^b^**	***1.955* ^a^**	***0.028***	**0.0001**	***64.19***
Gallic acid	0.047 ^c^	0.149 ^b^	0.497 ^a^	0.005	0.0001	98.14
Vanillic acid	0.130 ^c^	0.460 ^b^	0.905 ^a^	0.010	0.0001	74.52
Syringic acid	0.037 ^c^	0.100 ^b^	0.155 ^a^	0.009	0.0001	59.52
Hydroxybenzoic acid	0.060 ^c^	0.152 ^b^	0.293 ^a^	0.008	0.0001	46.36
Ellagic acid	0.020 ^c^	0.043 ^b^	0.099 ^a^	0.005	0.0001	62.46
Protocatechuic acid	0.001 ^b^	0.002 ^b^	0.007 ^a^	0.000	0.0001	44.15
						
***Hydroxycinnamic acids***	***1.075 ^c^***	***2.761*** **^b^**	***4.300* ^a^**	***0.106***	**0.0001**	***61.73***
Chlorogenic acid	0.222 ^c^	0.587 ^b^	0.984 ^a^	0.025	0.0001	45.18
Caffeic acid	0.017 ^c^	0.050 ^b^	0.087 ^a^	0.005	0.0001	68.48
Metoxicinnamic acid	0.189 ^c^	0.449 ^b^	0.714 ^a^	0.017	0.0001	62.43
Ferulic acid	0.536 ^c^	1.346 ^b^	2.092 ^a^	0.075	0.0001	70.78
Coumaric acid	0.110 ^c^	0.326 ^b^	0.412 ^a^	0.014	0.0001	47.05
Cinnamic acid	0.002 ^b^	0.003 ^b^	0.011 ^a^	0.000	0.0001	76.46
						
**Flavonoids**	**0.434 ^c^**	**0.989 ^b^**	**1.580 ^a^**	**0.042**	**0.0001**	**54.87**
***Flavanols***	***0.417 ^c^***	***0.951* ^b^**	***1.500* ^a^**	***0.042***	**0.0001**	***56.78***
Epigallocatechin	0.296 ^c^	0.658 ^b^	0.923 ^a^	0.025	0.0001	59.27
Catechin	0.015 ^b^	0.026 ^b^	0.054 ^a^	0.003	0.0001	59.00
Epicatechin	0.106 ^c^	0.267 ^b^	0.523 ^a^	0.018	0.0001	52.08
						
***Flavonols***	***0.017 ^c^***	***0.038* ^b^**	***0.080* ^a^**	**0.003**	**0.0001**	***52.95***
Rutin	0.015 ^c^	0.037 ^b^	0.077 ^a^	0.003	0.0001	50.09
Quercetin	0.001 ^b^	0.001 ^b^	0.003 ^a^	0.000	0.0001	55.81
						
**Stilbene**	**0.001 ^b^**	**0.002 ^b^**	**0.004 ^a^**	**0.000**	**0.0001**	**74.37**
Resveratrol	0.001 ^b^	0.002 ^b^	0.004 ^a^	0.000	0.0001	74.37

^1^ RI—recovery index. *p*-value represents the level of significance, and different letters within each row mean significant differences between samples.

**Table 5:** The antioxidant potential of blackcurrant extracts (fruits, leaves, and pomace).

	Fruits	Leaves	Pomace	SEM	*p*-Value
Phosphomolybdate assay (mM equiv ascorbic acid)	92.03 ^c^	472.1 ^a^	299.3 ^b^	7.529	0.0001
DPPH ^1^ (mM equiv Trolox)	235.6 ^b^	1041.6 ^a^	72.70 ^c^	7.191	0.0001
TEAC ^2^ (mM equiv Trolox)	86.33 ^b^	360.6 ^a^	34.17 ^c^	1.160	0.0001
Iron chelation capacity (mM equiv EDTA)	0.380 ^c^	2.450 ^a^	0.414 ^b^	0.020	0.0001

^1^ 1,1-Diphenyl-2-picrylhydrazyl, ^2^ Trolox Equivalent Antioxidant Capacity. *p*-value represents the level of significance, and different letters within each row mean significant differences between samples.

**Table 6:** The effect of blackcurrant extracts has the potential to delay in vitro-induced lipid peroxidation.

Samples	MDA ^1^, (mg/kg)
Fresh meat (unperoxidized)	0.568 ^d^
Fresh meat (in vitro peroxidized with Fe^3+^/AA system)	1.150 ^a^
Fresh meat (in vitro peroxidized with Fe^3+^/AA system, inhibited with BHT)	0.648 ^cd^
Fresh meat (in vitro peroxidized with Fe^3+^/AA system, inhibited with blackcurrant leaf extract)	0.714 ^c^
Fresh meat (in vitro peroxidized with Fe^3+^/AA system, inhibited with blackcurrant fruit extract)	0.880 ^b^
Fresh meat (in vitro peroxidized with Fe^3+^/AA system, inhibited with blackcurrant pomace extract)	0.951 ^b^
SEM	0.025
*p*-value	0.0001

^1^ Malondialdehyde. *p*-value represents the level of significance, and different letters within each row mean significant differences between samples.

## Data Availability

The original contributions presented in the study are included in the article, further inquiries can be directed to the corresponding author.
